# Designing Functionally Versatile, Highly Immunogenic Peptide-Based Multiepitopic Vaccines against Foot-and-Mouth Disease Virus

**DOI:** 10.3390/vaccines8030406

**Published:** 2020-07-22

**Authors:** Sira Defaus, Mar Forner, Rodrigo Cañas-Arranz, Patricia de León, María J. Bustos, Miguel Rodríguez-Pulido, Esther Blanco, Francisco Sobrino, David Andreu

**Affiliations:** 1 Departament de Ciències Experimentals i de la Salut, Universitat Pompeu-Fabra, 08003 Barcelona, Spain; sira.defaus@upf.edu (S.D.); mar.forner@upf.edu (M.F.); 2 Centro de Biología Molecular “Severo Ochoa” (CSIC-UAM), 28049 Madrid, Spain; rcannas@cbm.csic.es (R.C.-A.); pdeleon@cbm.csic.es (P.d.L.); mjbustos@cbm.csic.es (M.J.B.); mrrodriguez@cbm.csic.es (M.R.-P.); 3 Centro de Investigación en Sanidad Animal (CISA-INIA), Valdeolmos, 28130 Madrid, Spain; blanco@inia.es

**Keywords:** peptide-based vaccines, foot-and-mouth disease virus, multivalency, click chemistry, swine host

## Abstract

A broadly protective and biosafe vaccine against foot-and-mouth disease virus (FMDV) remains an unmet need in the animal health sector. We have previously reported solid protection against serotype O FMDV afforded by dendrimeric peptide structures harboring virus-specific B- and T-cell epitopes, and also shown such type of multivalent presentations to be advantageous over simple B-T-epitope linear juxtaposition. Chemically, our vaccine platforms are modular constructions readily made from specified B- and T-cell epitope precursor peptides that are conjugated in solution. With the aim of developing an improved version of our formulations to be used for on-demand vaccine applications, we evaluate in this study a novel design for epitope presentation to the immune system based on a multiple antigen peptide (MAP) containing six immunologically relevant motifs arranged in dendrimeric fashion (named B_2_T-TB_2_). Interestingly, two B_2_T units fused tail-to-tail into a single homodimer platform elicited higher B- and T-cell specific responses than former candidates, with immunization scores remaining stable even after 4 months. Moreover, this macromolecular assembly shows consistent immune response in swine, the natural FMDV host, at reduced dose. Thus, our versatile, immunogenic prototype can find application in the development of peptide-based vaccine candidates for various therapeutic uses using safer and more efficacious vaccination regimens.

## 1. Introduction

Foot-and-mouth disease (FMD), a highly contagious viral disease of cloven-hoofed animals, persists as a threat for livestock production in both endemic and allegedly “free” countries. The disease is currently present in regions of Africa, Middle East, and Asia. Among the methods recommended by the World Organization for Animal Health (OIE) for FMD control and eradication, chemically inactivated virus-derived vaccines are still in wide use [1,2,3]. However, such conventional vaccines have serious limitations such as (i) the risk of virus escape during vaccine preparation, (ii) extensive genetic and antigenic variability, illustrated by the existence of seven different serotypes and many variants, (iii) difficulties for serological distinction between infected and vaccinated animals (DIVA concept), and (iv) the requirement for cold chain logistics to preserve vaccine stability [4,5]. These drawbacks, together with the fatal consequences of FMDV sporadic but still severe outbreaks [6,7,8], evince the need for better FMD countermeasure policies. In this regard, subunit vaccines devoid of infectious agent, particularly those based on synthetic peptides, have arisen as an attractive alternative due to their multiple advantages including safety, marker nature, fine-tuning to different strains, and easy handling, shipping and storage [9,10].

Although peptides are able to replicate well-known antigenic epitope motifs [11,12] of an entire virus, early attempts of FMD vaccination using recognized linear B-cell peptide epitopes induced only limited protection in natural hosts [13,14,15,16]. A deeper understanding of the cellular and molecular mechanism underpinning immunogenic responses has contributed to the currently prevailing idea that the protective immune response to a vaccine is not only due to the presence of circulating antibodies via B-cell activation (humoral immunity), but also due to the actions of sensitized T-lymphocytes (cell-mediated immunity) [17,18,19,20]. In this context, T-cell epitopes efficiently recognized by T lymphocytes from different animals upon heterotypic FMDV infection have been identified, thus extending the repertoire of viral T-cell epitopes to be considered for FMDV subunit and synthetic vaccine strategies [21,22,23,24]. Apart from the inclusion of an adequate T-cell epitope in the vaccine formulation, T- and B-cell epitope orientation and multiplicity are also decisive factors for developing successful peptide-based vaccine candidates [25,26,27]. In tune with this, we first reported a dendrimeric peptide consisting of one copy of a T-cell epitope conjugated in branched fashion to four copies of a B-cell epitope (serotype C) [28] as capable of inducing high levels of protective mucosal IgA response in domestic pigs infected with FMDV [29]. Remarkably, a bivalent dendrimer version bearing the same T-cell epitope conjugated to two copies of the B-cell epitope (serotype O) elicited even better results [30], and a similar construct displaying B-cell epitopes from classical swine fever virus (CSFV) also showed humoral response [31] in pigs. These results supported the relevant cooperative role of the T-cell epitope inducing neutralizing response against different viruses. Subsequently, different ligation strategies were explored pointing out to a B_2_T platform made via maleimide conjugation as a highly valuable, cost-effective FMDV vaccine candidate providing full and longer protection of vaccinated animals after viral challenge [30,32]. 

Taken together, all these attempts along with other reported examples [33,34,35] reinforce multivalence as an extremely powerful concept having also a positive impact on the immunogenicity of synthetic vaccine prototypes. Indeed, dendrimer research has a strong impact on a broad range of biotech fields, as shown by the spectacular growth in the number of related publications [36]. While the potential applications of these multivalent presentations with their highly customizable properties are plentiful, some of their current uses relevant to vaccinology are still scarce. In this study, advances in the potential of spatially diverse architectures on the in vivo biological response of dendrimers as subunit vaccines against FMDV, as well as recent progress in design-driven optimization of this new generation of multiepitope bioconjugate vaccines against FMDV, are presented.

## 2. Materials and Methods

### 2.1. Reagents and Analytical Procedures

Fmoc-protected amino acids and HBTU were from Iris Biotech (Marktredwitz, Germany). Fmoc-Rink-amide ChemMatrix resin was from PCAS Bio-Matrix, Inc. (Saint-Jean-sur-Richelieu, QC, Canada). HPLC-grade CH_3_CN and peptide-synthesis-grade DMF, CH_2_Cl_2_, DIEA, and TFA were from Carlo Erba-SdS (Sabadell, Spain) and/or Fisher Scientific International, Inc. (Pittsburgh, PA, USA). All other reagents were of the highest quality commercially available from Sigma-Aldrich (Madrid, Spain). Analytical reversed-phase HPLC was performed on C18 columns (4.6 × 50 mm, 3 μm, Phenomenex, Torrance, CA, USA) in a model LC-2010A system (Shimadzu, Kyoto, Japan). Solvent A was 0.045% (*v*/*v*) TFA in water; solvent B was 0.036% (*v*/*v*) TFA in CH_3_CN. Elution was done with linear 20–60% gradients of solvent B into A over 15 min at 1 mL/min flow rate, with UV detection at 220 nm. Preparative HPLC was performed on C18 (10 × 250 mm, 10 μm, Phenomenex) in a Shimadzu LC-8A instrument. Solvents A and B were 0.1% TFA (*v*/*v*) in water and CH_3_CN, respectively, and elution was again with linear gradients of solvent B into A over 20 min, at 5 mL/min flow rate with UV detection at 220 nm. Fractions of satisfactory purity (>95%) by analytical HPLC were pooled and lyophilized. Purified peptides and conjugates were characterized for identity by ESI-MS with an LCMS-2010 EV mass spectrometer (Shimadzu, Kyoto, Japan) controlled using LabSolutions LCMS software and/or by MALDI-TOF MS in a ABSciex 4800 Plus spectrometer (Applied Biosystems, Foster City, CA, USA), using α-cyano-4-hydroxycinnamic acid as matrix. 

### 2.2. General Peptide Synthesis Procedures 

Linear peptides were assembled in a Prelude synthesizer (Protein Technologies, Inc., Tucson, AZ, USA) running Fmoc SPPS protocols at 0.1 mmol scale on Fmoc-Rink-amide ChemMatrix resin. The side chain functionalities were protected with tertbutyl (Asp, Glu, Ser, Thr, Tyr), tert-butyloxycarbonyl (Lys, Trp), NG-2,2,4,6,7-pentamethyldihydrobenzofuran-5-sulfonyl (Arg), and trityl (Asn, Gln, His) groups. Eight-fold excess of Fmoc-L-amino acids and HBTU, in the presence of a double molar amount of DIEA, were used for the coupling steps, with DMF as solvent. All peptides were fully deprotected and cleaved from the resin with TFA/H_2_O/triisopropylsilane (95:2.5:2.5 *v*/*v*, 90 min, r.t.), precipitated by addition of chilled diethyl ether, taken up in aqueous AcOH (10% *v*/*v*), and lyophilized. Reverse-phase HPLC purification gave homogeneous materials with the expected mass by LC-MS.

### 2.3. Functionalization and Conjugation of Peptides 

**Figure 1:**
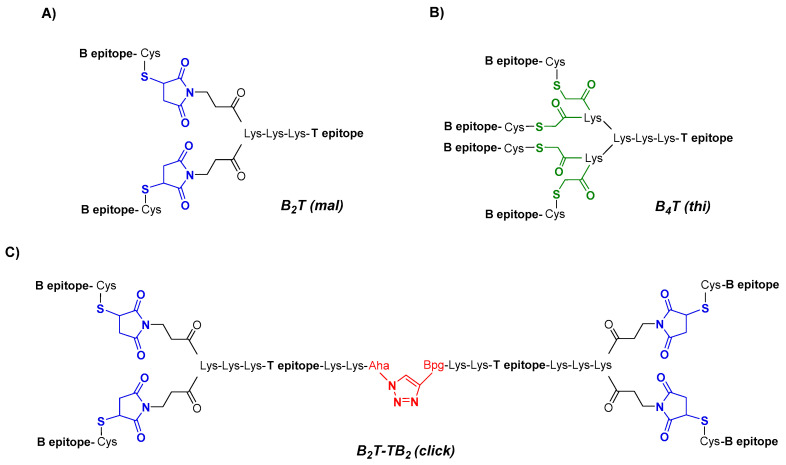
Conjugation approaches to dendrimeric immunogens based on lysine-branched constructs containing B- and T-cell epitopes of foot-and-mouth disease virus (FMDV) O UKG/11/2001 in a 2:1 (**A**), 4:1 (**B**) or 4:2 (**C**) ratio; the Lys-Lys motif in each construct is a putative cathepsin D cleavage site. Different chemical ligations strategies were employed to connect the epitopes, including maleimide (blue), thioether (green), and triazole (red) linkages.

Peptides reproducing the B- and T-cell epitopes of FMDV O UKG/11/2001 in different arrangements are shown in Figure 1. All peptides were made from precursors prepared by solid phase synthesis and purified prior to conjugation as previously specified. In all cases, branching was achieved by T epitope N-terminal elongation with two Lys units followed by either three [B_4_T(thi)] or one [B_2_T(mal)] additional Lys in a branched arrangement. To allow conjugation, T epitope was functionalized with chloroacetyl [B_4_T(thi)] or maleimido [B_2_T(mal)] groups, and an *N*-acetylated B epitope with a C-terminal Cys whose free thiol group reacts with either chloroacetyl or maleimide units. For B_2_T-TB_2_(click) synthesis, tail-to-tail fusion of two units of B_2_T(mal) peptide was accomplished by further functionalization of T epitope at the C-terminal with either an azide- or an alkyne-containing non-canonical amino acid and those end groups finally reacting via CuAAC. Additional details on the synthesis are available in refs [30,37]. The final products were purified to near homogeneity by HPLC and characterized by MS. 

### 2.4. Animal Immunizations 

The immune response to B_2_T(mal), B_4_T(thi), and B_2_T-TB_2_(click) dendrimeric peptides was assessed in outbred Swiss ICR-CD1 mice (Harlan Laboratories, Boxmeer, The Netherlands) and/or domestic Landrace X Large White pigs (Agropardal SL, Almendros, Cuenca, Spain), free of antibodies against FMDV. Animals were maintained under standard housing conditions in the CBMSO (mice) and Departamento de Reproducción Animal, INIA (pigs). Animal facilities and experimental procedures were conducted in accordance with protocols approved by CSIC and INIA Committees on Ethics of Animal Experiments and Biosafety, as well as of the National Committee on Ethics and Animal Welfare (PROEX 034/15 at CBMSO; PROEX 218/14, PROEX 47/13 and PROEX 214/15 at INIA). 

Female mice (5–9 weeks old) in groups of five received a primary immunization (day 0) followed by a booster dose on day 21, by subcutaneous route, with 100 μg of each conjugated dendrimeric peptide emulsified in Montanide ISA-50 V2 (Seppic, Puteaux, France). Serum samples were collected by mandibular bleeds on days 0, 21, and 40. The animals were humanely sacrificed on day 40.

Two month-old pigs were randomly assigned to three groups of four animals each. The pigs received a primary immunization (day 0) followed by a booster dose on day 39, by intramuscular route with 2 mL of an emulsion containing 2 or 0.5 mg of dendrimer B_2_T(mal) or B_2_T-TB_2_(click) in Montanide oil ISA-50 V2. Two additional pigs inoculated with a mock emulsion prepared using PBS and adjuvant were kept as infection controls. Blood samples were collected at days 0, 7, 14, 21, 28, 91, 127, and 154 pi to obtain serum and peripheral blood mononuclear cells (PBMCs). 

### 2.5. Detection of Specific Anti-FMDV Antibodies by ELISA 

Total anti-FMDV antibodies were determined by ELISA. Briefly, 96-well plates (Nunc) were coated with B peptide (1 µg) overnight at 4 °C. Duplicate three-fold dilution series of each serum sample were prepared in 50 µL, starting at 1/100. Pre-immune sera from peptide-immunized animals and sera from non-immunized animals were used as negative controls. Specific antibodies were detected with HRP-conjugated protein A (Thermo Fisher, Waltham, MA, USA), diluted 1/4000. Color development was obtained after addition of 100 µL/well of TMB (Sigma Aldrich) and stopped by an equal volume of H_2_SO_4_ 1M. Plates were read at 450 nm and titers in a log10 scale were expressed as the reciprocal of the last dilution giving the absorbance recorded in the control wells (serum at day 0) plus 2 SD.

### 2.6. Virus Neutralization Test (VNT)

Serial two-fold dilutions of sera were incubated with 100 infection units—50% tissue culture infective doses (TCID50)— of FMDV O UKG/11/2001, for 1 h at 37 °C. Then, a cell suspension of IBRS-2 cells in DMEM was added and plates were incubated for 72 h. Monolayers were controlled for development of cytopathic effect (cpe), fixed and stained. End-point titers in a log10 scale were calculated as the reciprocal of the serum dilution that neutralized FMDV infection in 50% of the wells [30].

### 2.7. PBMCs Isolation and IFN-γ Detection by ELISPOT

Porcine peripheral blood mononuclear cells (PBMCs) were isolated by density gradient centrifugation using Histopaque-1077 (Sigma-Aldrich, St. Louis, MO, USA). Cell counting and viability were tested by trypan blue staining. For the IFN-γ ELISPOT assay 2.5 × 10^5^ PBMCs were shed in triplicate wells of Immobilon-P plates (Merck Millipore, Madrid, Spain) coated with 5 µg/mL of anti-pig IFN-γ antibody (clone P2G10, BD Biosciences, San Agustín de Guadalix, Madrid, Spain). For in vitro antigen recall, PBMCs were stimulated with 50 μg/mL of the peptide used for pig immunization [38]. As positive control, PBMCs were incubated with 10 μg/mL of phytohaemagglutinin (Sigma-Aldrich, St. Louis, MO, USA) using cells incubated without antigen as negative control. After 48 h at 37 °C—5% CO_2_, plates were washed and incubated with 2 µg/mL of biotinylated anti-mouse IFN-γ antibody (clone P2C11, BD Biosciences) and HRP-streptavidin (BD Biosciences). Antibody was visualized with 3-amino-9-ethyl carbazole (BD Biosciences). The frequency of peptide-specific T-cells was expressed as the mean number of spot-forming cells/10^6^ PBMCs, with background values (number of spots in negative control wells) subtracted from the respective counts of stimulated cells. 

### 2.8. Statistical Analyses

Differences among peptide-immunized groups in FMDV-antibody titers and number of IFN-γ producing cells, were analyzed by one-way ANOVA, followed by Tukey’s post-hoc comparisons tests. Values are cited in the text as means ± SD. All *p* values are two sided, and *p* values < 0.05 were considered significant. Statistical analyses were conducted using GraphPad Prism Software 5.0 (San Diego, CA, USA).

## 3. Results and Discussion

### 3.1. Dendrimeric Peptide-Based FMD Candidate Vaccine Preparation 

Three different multivalent constructs (i.e., B_2_T(mal), B_4_T(thi) and B_2_T-TB_2_(click)) were synthesized using a branched lysine core matrix from which various arrangements exploiting both alpha (α) and epsilon (ε) reactive amino groups in Lys residues were implemented. Although with different scaffold presentations, all dendrimers in this study were composed of linear peptide modules replicating either B- or T-cell relevant epitopes derived from FMDV serotype O UKG/11/2001, specifically the VP1(140–158) and 3A(21–35) sequences (Table 1). In all constructs a Lys-Lys dipeptide motif was included to define a cleavage site for cathepsin D, a protease putatively involved in in vivo antigen processing for presentation to the MHC class II molecules [39]. Given the different frameworks designed, several linking functionalities were used at various parts of the peptide building blocks, to carry out the corresponding conjugation strategies (Figure 1). 

#### 3.1.1. Bivalent-Branched B_2_T Conjugate [B_2_T(mal)] 

A practical route to B_2_T-type immunogens was achieved through thiol–maleimide chemistry (Figure 1A) [30]. Briefly, two C-terminally thiol-functionalized B-cell epitope branches were connected via maleimide linkages at both α- and ε-amino ends of a branched Lys core T-epitope. This allowed an efficient conjugation chemistry, optimally run at pH 6, where thiol oxidation was essentially averted. The total absence of dimerization made possible a strictly stoichiometric use of thiol-functionalized peptide B epitope that, combined with fast reaction times and minimal by-product formation, led to very pure end products in a highly efficient fashion [40].

#### 3.1.2. Tetravalent-Branched B_4_T Conjugate [B_4_T(thi)]

The synthetic approach chosen for this construct was based on the chemoselective thioether ligation (Figure 1B) [41] of (i) the T-cell epitope, N-terminally elongated with two (cathepsin D site) plus three additional Lys residues defining a tetravalent dendrimeric core (the last two Lys residues with both α- and ε-amino groups functionalized as 2-chloroacetyl derivatives); and (ii) four copies of the 19-residue VP1 B-cell epitope, acetylated at the N-terminus and C-terminally elongated with a Cys residue. While the two separately made precursors were available in highly pure form by solid phase peptide synthesis, the final thioether-based conjugation at pH 7 was slow and unselective, requiring extensive purification to give an HPLC-homogeneous product as described earlier [29].

#### 3.1.3. Dimeric B_2_T-TB_2_ Conjugate [B_2_T-TB_2_(click)] 

A further step into chemically well-defined, single molecule vaccine platforms displaying a high number of relevant peptide motifs arranged in a dendrimeric fashion was devised by tail-to-tail fusion of two B_2_T maleimide subunits via orthogonal chemical ligation by copper(I)-catalyzed azide–alkyne 1,3-cycloaddition (CuAAC), leading to a novel B_2_T-TB_2_ multivalent platform [37] (Figure 1C). Our synthesis strategy involved preparation of functionalized peptide building blocks based on the B_2_T(mal) predecessor but with site-specific modifications such as an extra C-terminal functionalization of the T-cell epitope with either azide- or alkyne-containing non-canonical amino acids to enable final subunit assembly. Full details on the stepwise click chemistry-based approach, including the solving of various adverse issues encountered during process optimization due to size and structural complexity of the building blocks have been extensively reported [37].

### 3.2. Relevance of Dendrimer Scaffold Architecture to Immunogenicity 

**Figure 2:**
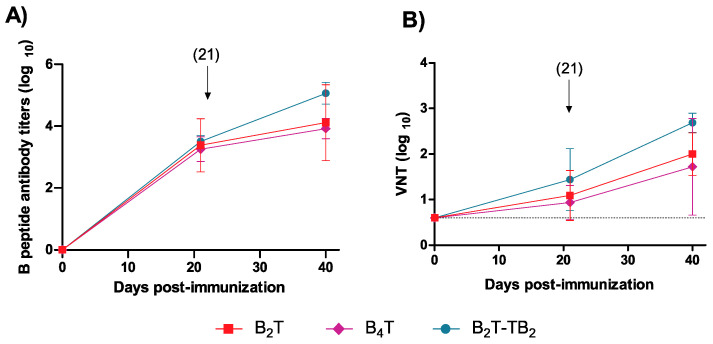
Anti-FMDV antibody responses of mice vaccinated with conjugates B_2_T(mal), B_4_T(thi), and B_2_T-TB_2_(click). (**A**) ELISA-determined anti-B peptide response in sera collected at the indicated days post-immunization. Each point depicts mean antibody titers ± SD for each group. Arrow shows the day of boost. (**B**) Neutralizing (VNT) antibody responses at days 21 (pre-boost) and 40 (post-boost) pi. Dotted line indicates the detection limit. Each symbol represents the value for an individual mouse. Horizontal lines indicate the geometric mean for each animal group. No individual spontaneous reactivity was observed in the titers determined at day 0. Differences were not statistically significant.

To explore the impact of particular assembly patterns on immunogenic behavior, specific anti- FMDV antibodies were determined by ELISA in sera at days 0, 20 (first dose), and 40 (second dose) post-immunization (pi) from mice immunized with the synthetic dendrimeric conjugates depicted in Figure 1. All peptides elicited consistent, comparable IgG titers [3.7 ± 0.4, 3.2 ± 0.4 and 3.5 ± 0.2 for B_2_T(mal), B_4_T(thi), and B_2_T-TB_2_(click), respectively] after the first dose (Figure 2A). Titers were boosted up, with some differences among groups, after a second dose. The largest increase in anti-FMDV titers after boost was recorded in mice given the B_2_T-TB_2_(click) immunogen (5.1 ± 0.3). The other two groups showed lower post-boosting titers: 4.6 ± 0.4 [B_2_T(mal)] and 3.9 ± 0.3 [B_4_T(thi)]. Therefore, in general terms B_2_T-TB_2_(click) dimer appears to elicit more anti-FMDV antibodies than its B_2_T(mal) constituent or the other tetravalent construct, B_4_T(thi). Likewise, after boosting, significant virus neutralizing titers (VNT) were obtained at day 40 pi in sera from all mice (Figure 2B). Again, as with IgG titers above, B_2_T-TB_2_(click) showed higher VNT values (2.7 ± 0.2) compared to the two other constructs: 2.0 ± 0.5 [B_2_T(mal)] and 1.7 ± 1.0 [B_4_T(thi)].

These results with three different conjugates clearly demonstrate that size, chemistry, flexibility, shape, and architecture play a crucial role, especially when considering in vivo immunogenicity of vaccine candidates whose structural differences may induce changes in the immunogen presentation and distribution, which in turn are responsible for their biological activity. These results are in agreement with previous work reporting that multimerization of B-cell epitopes enhances the responsiveness of B-cells for subsequent antibody production [42]. In terms of anti-FMDV immune response, the most active immunogen is the B_2_T-TB_2_(click) construct, whose particular three-dimensional branched structure may favor a suitable arrangement that maximizes multivalent interactions with cells involved in the immune response. In sum, our work shows how subtle changes in the spatial arrangement of constructions displaying identical epitope motifs, and even maintaining the same B-cell epitope multiplicity (i.e., B_4_T(thi) vs. B_2_T-TB_2_(click)), may substantially modify the biological response.

### 3.3. Immunogenicity of B_2_T-TB_2_ in Pigs: Dose Effect and Long-Term Response 

**Figure 3:**
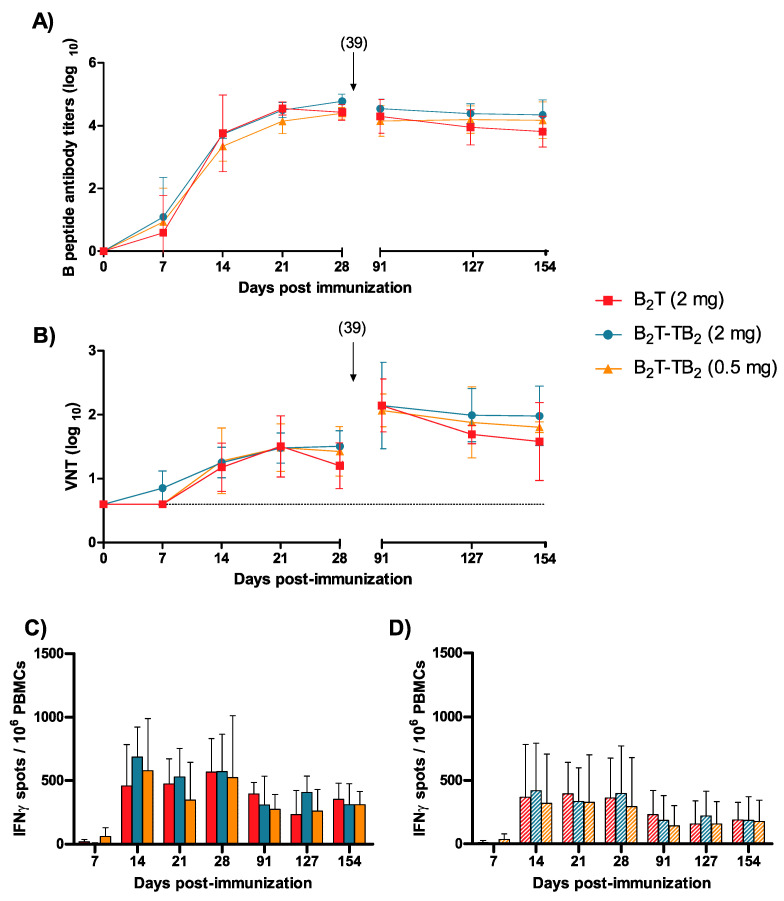
Long-term response and dose effect of B_2_T-TB_2_(click) dimer. Time-course of the specific immune responses against FMDV in pig sera collected on the indicated days post-immunization (7, 14, 21, 28, 91, 127, and 154 pi). (**A**) Antibody response analyzed by ELISA. Each point depicts mean antibody titers (calculated as described in Experimental Section) ± SD for each group of pigs. (**B**) Virus neutralization titers expressed as the reciprocal log10 of the last serum dilution that neutralized 100 TCID50 of FMDV O UKG/11/2001. Each symbol represents the value for an individual pig. Horizontal lines indicate the geometric mean for each animal group. Differences were not statistically significant. Arrows show the day of boost and dotted line indicates the detection limit. (**C**,**D**) Specific T-cell responses measured by an ex vivo IFN-γ ELISPOT. IFN-γ released by PBMCs from pigs stimulated in vitro with the corresponding dendrimer (solid bars) or with T-epitope (striped bars), respectively. The frequency of FMDV-specific IFN-γ secreting cells was determined as detailed in Experimental Section.

To evaluate the long-term immune response elicited by our novel B_2_T-TB_2_(click) construct in a comparable way to the well-established B_2_T(mal) vaccine prototype [32], domestic pigs in three different groups of four animals each were immunized and boosted with 2 mg—the same amount used in previous studies—of B_2_T(mal) or B_2_T-TB_2_(click), and 0.5 mg of B_2_T-TB_2_(click), a four-times lower dose. Two control pigs were inoculated with PBS (data not shown). In this vaccination trial, the booster was delayed to day 39 pi, to evaluate in more detail the early response. Specific anti-FMDV antibodies were determined by ELISA in pig sera at different days post-immunization. A consistent, long-lasting response was observed in all the immunized animals from day 14 pi (Figure 3A). Antibody titers in all tested groups were high, reaching ca. 4 log units, and were maintained up to day 154 pi (5 months pi). Both 2 and 0.5 mg doses of B_2_T-TB_2_ (click) peptide elicited consistent and comparable IgG titers at early days [3.7 ± 0.1 and 3.4 ± 0.5, respectively at day 14 pi], remaining similar in both B_2_T-TB_2_(click) immunized groups longitudinally [4.3 ± 0.5 (2 mg) and 4.2 ± 0.6 (0.5 mg) at day 154 pi] (Figure 3A). No specific anti-FMDV antibodies were detected in control non-immunized animals (data not shown).

Significant virus neutralization test (VNT) titers were also noticed from day 14 pi, with an increase observed in all animals upon boosting (day 39 pi) (Figure 3B). Interestingly, by day 154 pi, average VNT titers similar to those observed before boost were detected (~17 weeks post-booster). As expected, control animals did not show any specific humoral response. Taken together, these results demonstrate that administration of a reduced 0.5 mg dose of our novel B_2_T-TB_2_(click) conjugate can elicit in swine long-lasting FMDV-neutralizing antibodies and consistent T-cell responses.

The specific T-cell responses elicited by B_2_T(mal) peptide and its homodimer B_2_T-TB_2_(click), at several days pi were determined by ELISPOT analysis of the IFN-γ- expressing PBMCs. High frequencies of spot-forming cells were found already at day 14 pi in pigs immunized with both dendrimers at the same 2 mg dose, in response to in vitro recall with the corresponding B_2_T(mal) or B_2_T-TB_2_(click) peptides [458.3 ± 326.2 and 687.3 ± 236.2, respectively] (Figure 3C). Remarkably, significant responses were still observed in all immunized animals upon boost at day 39 pi, and up to day 154 pi. On average, pigs given B_2_T-TB_2_(click) showed higher frequencies of IFN-γ-expressing PBMCs than those receiving just B_2_T(mal) over the long assay timespan, even using a reduced dose of 0.5 mg. Moreover, PBMC stimulations with the T-epitope peptide alone (i.e, not elongated with any Lys core) paralleled those observed with the corresponding dendrimer constructs (Figure 3D), confirming the recognition of 3A(21–35) as a T-cell epitope by porcine lymphocytes. All the responses were specific, as no peptide-driven IFN-γ-producing cells were detected in the nonimmunized pigs (data not shown).

## 4. Conclusions

In this study we have assessed the close relationship between dendrimer architecture and biological response in a realistic infectious disease scenario by means of in vivo animal studies with multi-epitope peptide vaccines against FMDV. We have also reported on the main aspects [discussed in further detail in [37]] of the refinement of our synthetic methodologies and their biological impact. Specifically, dendrimers bearing same B- and T-cell epitopes from FMDV O UKG/11/2001 (an isolate belonging to FMDV/Type O/PanAsia-1 topotype responsible for the pandemic in Asia, extended to parts of Africa and Europe in 1998–2001), in different arrangements and made by diverse conjugation approaches, were explored in search of an improved FMDV vaccine candidate. These include: (i) our original tetravalent B_4_T(thi) candidate with four copies of the B-epitope and one T-epitope centrally attached to a lysine core matrix by conventional thioether ligation; (ii) the optimized B_2_T(mal) prototype, a downsized version relative to B_4_T, with only two copies of the B-cell epitope attached to the T-epitope through maleimide linkages; this is shown as particularly advantageous, not only in terms of immune response but also regarding chemical simplicity, conjugation reaction rates and yields; and (iii) the B_2_T-TB_2_(click) construct, a vaccine platform of substantial size (~120 residues, MW ~14.5 kDa) providing tetra- and bivalent display of B- and T-cell epitopes, respectively, achieved by a combination of thiol-maleimide ligation and CuAAC cycloaddition. All these immunogens were administered first in mice and shown capable of inducing specific total IgGs and neutralizing antibodies in different levels. Besides, the immune response and protection elicited by our novel B_2_T-TB_2_(click) construct was studied in detail in pigs, a main natural FMDV host, with regard to the induction of long-term protective immune responses as well as the potential of reducing peptide dose, both important requirements for an efficient vaccine candidate. While these experiments were performed using outbred domestic pigs with different individual genetic backgrounds, the levels of animal-to-animal variation did not exceed those observed in other related studies [32].

This is, as far as we know, one of very few comparative studies on multivalent presentations of peptide epitopes from FMDV to the immune system [43,44,45,46]. In this work, all dendrimeric versions of the immunogens tested elicited antibodies against FMDV O UKG/11/2001. Interestingly, we have observed a trend toward higher antibody titers in animal groups immunized with our new homodimer B_2_T-TB_2_(click). Furthermore, we have shown that these high titers of neutralizing antibodies, which can be considered as protective according to previous results with B_2_T immunized and challenged pigs [32,47], can be maintained at long term. Moreover, less amount of B_2_T-TB_2_(click) immunogen can elicit consistent anti-FMDV immune responses, reducing the possible costs associated with vaccine manufacturing. Therefore, this functionally versatile and highly immunoeffective peptide-based multiepitopic vaccine defines a valuable approach to vaccine candidates with multiple applications based on similar architectures. Further studies are under way to confirm the protective responses in pigs after viral challenge, as well as to elucidate the structure–function relationships of these dendritic bioconjugates and their immunogenicity. That will allow for a high degree of control over scaffold geometry that can help develop candidates with optimized architecture, facilitate targeted delivery, and improve efficacy of peptide-based subunit vaccines.

## Figures and Tables

**Table 1:** Peptide-based vaccine candidates.

General Name	B_2_T (Mal)	B_4_T (Thi)	B_2_T-TB_2_ (Click)
General structure ^a^	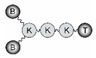	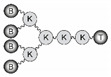	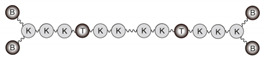
MW ^b^	6742.8 Da	11,204.1 Da	14,247.6 Da
HPLC ^c^	6.9 min (98%)	7.1 min (95%)	7.9 min (97%)
B epitope	acetyl-PVTNVRGDLQVLAQKAARTC-amide		
T epitope	AAIEFFEGMVHDSIK-amide		

^a^ B_n_T_n_ construct with n B epitope copies linked to a T epitope in different dendrimeric architectures. ^b^ Experimental peptide mass obtained by LC/MS. ^c^ Retention time on a C18 column (Luna, 4.6 mm × 50 mm, 3 mm; Phenomenex) eluted with a 20–60% linear gradient of solvent B (0.036% TFA in MeCN) into solvent A (0.045% TFA in H_2_O) over 15 min. In parenthesis, HPLC homogeneity of purified material.
